# CRISPR‐Cas9–Mediated Genome Editing Confirms *EPDR1* as an Effector Gene at the BMD GWAS‐Implicated ‘*STARD3NL*’ Locus

**DOI:** 10.1002/jbm4.10531

**Published:** 2021-07-23

**Authors:** James A Pippin, Alessandra Chesi, Yadav Wagley, Chun Su, Matthew C Pahl, Kenyaita M Hodge, Matthew E Johnson, Andrew D Wells, Kurt D Hankenson, Struan F A Grant

**Affiliations:** ^1^ Center for Spatial and Functional Genomics Children's Hospital of Philadelphia Philadelphia PA USA; ^2^ Department of Orthopedic Surgery University of Michigan Medical School Ann Arbor MI USA; ^3^ Genetics and Molecular Biology Graduate Program, Laney Graduate School Emory University Atlanta GA USA; ^4^ Department of Pathology and Laboratory Medicine University of Pennsylvania Perelman School of Medicine Philadelphia PA USA; ^5^ Department of Pediatrics University of Pennsylvania Perelman School of Medicine Philadelphia PA USA; ^6^ Divisions of Genetics and Endocrinology Children's Hospital of Philadelphia Philadelphia PA USA

**Keywords:** BONE MINERAL DENSITY (BMD), CHROMATIN CAPTURE, CRISPR, OSTEOBLAST DIFFERENTIATION, OSTEOBLAST PROGENITOR

## Abstract

Genome‐wide–association studies (GWASs) have discovered genetic signals robustly associated with BMD, but typically not the precise localization of effector genes. By intersecting genome‐wide promoter‐focused Capture C and assay for transposase‐accessible chromatin using sequencing (ATAC‐seq) data generated in human mesenchymal progenitor cell (hMSC)‐derived osteoblasts, consistent contacts were previously predicted between the *EPDR1* promoter and multiple BMD‐associated candidate causal variants at the ‘*STARD3NL*’ locus. RNAi knockdown of *EPDR1* expression in hMSC‐derived osteoblasts was shown to lead to inhibition of osteoblastogenesis. To fully characterize the physical connection between these putative noncoding causal variants at this locus and the *EPDR1* gene, clustered regularly interspaced short‐palindromic repeat Cas9 endonuclease (CRISPR‐Cas9) genome editing was conducted in hFOB1.19 cells across the single open‐chromatin region harboring candidates for the underlying causal variant, rs1524068, rs6975644, and rs940347, all in close proximity to each other. RT‐qPCR and immunoblotting revealed dramatic and consistent downregulation of *EPDR1* specifically in the edited differentiated osteoblast cells. Consistent with *EPDR1* expression changes, alkaline phosphatase staining was also markedly reduced in the edited differentiated cells. Collectively, CRISPR‐Cas9 genome editing in the hFOB1.19 cell model supports previous observations, where this regulatory region harboring GWAS‐implicated variation operates through direct long‐distance physical contact, further implicating a key role for *EPDR1* in osteoblastogenesis and BMD determination. © 2021 The Authors. *JBMR Plus* published by Wiley Periodicals LLC on behalf of American Society for Bone and Mineral Research.

## Introduction

BMD is a key clinical measure used to assess development and risk of the age‐related disease, osteoporosis.^(^ ^1^ ^)^ Low BMD is associated with a risk of bone fracture, including low trauma events in patients with osteoporosis.^(^ ^2^ ^)^ BMD is highly heritable, with genome‐wide–association studies (GWASs) having already identified hundreds of loci associated with disease risk in both adults^(^ ^1^, ^3^ ^)^ and children.^(^ ^4^, ^5^ ^)^ Although some progress has been made in recent years with the development of new methods to treat osteoporosis,^6^, ^7^ ^)^ functional investigation of BMD loci identified by GWASs should reveal target genes that represent potentially novel therapeutic avenues for prevention and treatment of this debilitating disease.

Often the sentinel SNP identified in a GWAS is not the causal SNP, but instead a proxy SNP in close linkage disequilibrium (LD) turns out to be the actual causal SNP.^(^ ^8^ ^)^ We recently published a high‐resolution variant‐to‐gene mapping analysis at BMD GWAS loci in a disease‐relevant cellular context: human mesenchymal stem‐cell–derived osteoblasts (hMSCs).^(^ ^9^ ^)^ We placed two constraints on our data derived from hMSCs differentiated into osteoblasts: first that SNPs in strong LD with a given GWAS sentinel variant must be accessible as determined by assay for transposase‐accessible chromatin using sequencing (ATAC‐seq), and second that these accessible BMD SNPs must be in direct physical contact with an accessible promoter as determined by high‐resolution DpnII‐based promoter Capture‐C covering 36,691 baited regions across the entire genome. Applying this epigenomic filter to DEXA‐derived and heel ultrasound‐associated signals highlighted 46 BMD GWAS loci and their putative effector gene targets.^(^ ^9^ ^)^ Some of these genes (*SMAD3*, *SMAD9*, *SPP1*, *WLS*, *FRZB*, *NOG*, and *MIR31HG*) are known regulators of bone osteogenesis, confirming the validity of our approach, whereas our data also implicated genes not previously studied in the context of bone metabolism.

In that initial study, *EPDR1* (encoding ependymin related protein 1) was one of the implicated genes we examined and found an influence on both osteoblastogenesis and adipogenesis. The *EPDR1* gene resides at the ‘*STARD3NL’* locus (sentinel SNP: rs6959212; minor allele frequency: ~35%), and downregulation of its expression via RNA interference (RNAi) in BMP2‐induced hMSC‐derived osteoblasts revealed a decrease in both alkaline phosphatase (ALP) and Alizarin Red S (ARS) staining, two fundamental measurements of osteoblastogenesis.^(^ ^9^ ^)^ Further analysis revealed a reciprocal role for *EPDR1* during adipogenic differentiation of the same hMSCs. Silencing of *EPDR1* increased the number of lipid droplets during adipogenic differentiation of hMSCs, which was accompanied by increased expression of *C/EBP*‐*α* and *PPAR*‐*γ*, two key adipogenic transcription factors (TFs).^(^ ^9^ ^)^

**Fig. 1:**
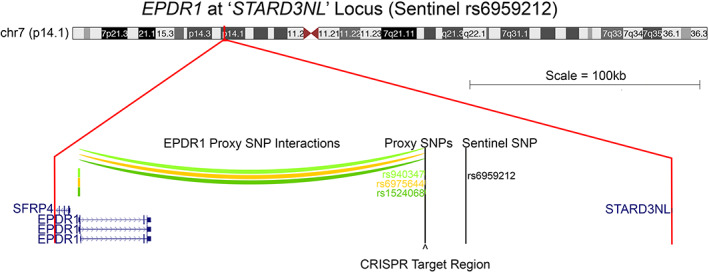
Promoter capture C interactions between the *EPDR1* gene promoter and proxy SNPs. rs1524068 (r^2^ = 1.0, dark green), rs6975644 (r^2^ = 0.553249, orange), and rs940347 (r^2^ = 0.995712, light green) located at the ‘*STARD3NL*’ locus (sentinel SNP rs6959212). Chromatin contacts between the proxy SNPs and the *EDPR1* gene promoter are approximately 150 kb (color arcs) and located in open chromatin regions. The 204‐bp clustered regularly interspaced short‐palindromic repeat Cas9 endonuclease (CRISPR‐Cas9) target region is indicated below the proxy SNPs with an arrowhead. All genes in the ‘*STARD3NL*’ locus are indicated at scale.

Although our RNAi approach provided valuable insight into the potential role of *EPDR1* in osteoblast differentiation, it did not prove a direct regulatory connection between the GWAS‐implicated proxy SNPs and this putative effector gene. To determine whether the BMD‐associated tight proxy‐SNP cluster resides in a cis‐regulatory element for the connected *EPDR1* gene, we sought to delete the open chromatin region (OCR) harboring the three key proxy SNPs to the sentinel (which are in strong LD and in very close proximity) using clustered regularly interspaced short palindromic repeat Cas9 endonuclease (CRISPR‐Cas9)–mediated gene editing (Fig. 1). We have successfully applied a comparable approach for the *TCF7L2* locus (rs7903146) associated with type 2 diabetes mellitus.^(^ ^10^ ^)^ In that study, we leveraged CRISPR‐Cas9–mediated deletion of a region immediately surrounding the putative causal variant that resulted in a 30‐fold loss in *ACSL5* expression. Thus, by removing the immediate region harboring the causal SNP, this approach implicated a functional linkage between the putative regulator harboring *TCF7L2* locus and the *ASCL5* gene.^(^ ^10^ ^)^

Although CRISPR‐Cas9–based genetic alteration has proven to be an efficient way to explore the functional relationship between long‐distal regulators (those not in close proximity to the target gene promoter) and effector genes, it is known to be challenging to execute this approach in primary hMSCs, given their lack of proliferation ability. Here, we selected an alternative cell model, an immortalized human fetal osteoblastic cell line (hFOB1.19) to pursue characterization of regulatory regions for human osteoblastogenesis. The hFOB1.19 cell line is easily passaged and expanded, a key requirement for CRISPR targeting. It contains a temperature‐sensitive mutant, tsA58, of the SV40 large T antigen that allows for the genome‐edited cells to proliferate under permissive conditions (33.5°C), and to subsequently differentiate into osteoblasts at a higher temperature (39.5°C).^(^ ^11^ ^)^ This allowed us to fully investigate the variant‐to‐gene contact between the proxy SNPs and the *EPDR1* gene promoter at the ‘*STARD3NL’* locus at various stages of differentiation.

## Materials and Methods

### Cell culture

hFOB1.19 and 293 T cells were cultured in the recommended media and maintained using standard culture conditions at 33.5°C and 37°C, respectively. Differentiation of hFOB1.19 cells into mature osteoblasts was accomplished by growing the cells at 39.5°C for 5 to 7 days for all experiments (see Supplementary Detailed Materials and Methods).

### RNAi treatment

Cells were seeded in 12‐well plates, and RNAi transfections were carried out the next day using sets of four ON‐TARGETplus RNAis (see Supplementary Table S1) according to the manufacturer's instructions. Twenty‐four hours later, media were replaced with fresh growth media. Cells designated for differentiation into mature osteoblasts were moved into a 39.5°C environment the following day; the cells were allowed to grow until assayed for ALP staining after 5 days (see Supplementary Detailed Materials and Methods for details).

### CRISPR constructs, lentivirus production, and hFOB1.19 infection

The synthesized single‐guide RNAs (sgRNAs) were cloned into the LentiCRISPRv2‐mCherry construct using a modified Golden Gate assembly method^(^ ^12^, ^13^, ^14^ ^)^ (see Supplementary Table S1). Proper insertion of the sgRNAs into the construct was confirmed using Sanger sequencing, and a pool containing an equal molar ratio of sgRNA constructs was generated for transfection. Then 293 T cells were seeded and allowed to adhere for 24 hours. Cells were transfected with equal molar ratios of LentiCRISPRv2‐mCherry construct, packaging construct, and envelope construct. Transfection media were replaced with growth media after 5 hours. Growth media containing the lentivirus were collected after 48 hours, filtered, and stored at –80°C until infection of hFOB1.19 cells.

Freshly thawed hFOB1.19 cells were plated and allowed to adhere for 24 hours. Growth media were replaced with fresh growth media, filtered lentivirus, and Polybrene to allow for lentiviral infection. Media were replaced after 72 hours, and cells were checked for expression of mCherry (see Supplementary Fig. S1). Lentiviral transduction efficiency was estimated by cell counting of both bright field live and mCherry‐positive cells. Cells were split post infection into freezer stocks and experimental plates to allow for assaying the cells at an early passage and as close to the original cells as possible (see Supplementary Detailed Materials and Methods).

### Multiplex sequencing

Genomic DNA from CRISPR‐edited plates was extracted, quantitated, and normalized. The proxy‐SNP target region was amplified by PCR (see Supplementary Fig. S2 and SupplementaryTable S1) in three concurrent reactions. The final PCR reaction contained pooled sequencing primers approximately 50 base pairs (bp) upstream (US) of each CRISPR cut site to provide sequence coverage for all possible CRISPR‐cas9–splicing sites. To eliminate primer carryover, each of the PCR reactions was followed by a purification step. Libraries were indexed and purified then checked on a Bioanalyzer 2100 (Agilent) for quality before being pooled. Finally, the libraries were sequenced on the MiSeq System (Illumina).

Sequencing libraries were first mapped to human genome assembly hg19 using BLAT. The spliced reads were extracted if the splicing junction within the read was larger than 2 bp and the rest of the read‐alignment ratio was above 95%. Spliced reads were further assigned to sgRNA primer‐pair sequences if the splicing site was within 60 bp of the CRISPR‐cas9 editing sites. The efficiency of each pair of sgRNAs was calculated independently for each library as the ratio of spliced reads located around their editing sites to total mapped reads. Finally, deletions for each pair of sgRNAs were visualized using ggplot2 (version 3.1.0) in R (see Supplementary Detailed Materials and Methods).

### Alkaline phosphatase assay

ALP staining was assessed as described in previous studies with hMSCs.^(^ ^9^ ^)^ Briefly, CRISPR‐edited hFOB1.19 cells were seeded in two 12‐well plates and allowed to adhere for 24 hours. The following day, one plate was moved to a 39.5°C environment and allowed to differentiate for 5 days, while the other remained in a 33.5°C environment. On the day of the ALP assay, fresh fixation and staining mixtures were prepared, media were removed, and cells were washed with Dulbecco's phosphate‐buffered saline. Cells were fixed in the plate, washed with ultrapure water, and staining solution was applied to the cells until the development of color. Once staining was complete, cells were washed with ultrapure water and allowed to air dry. Plates were photographed and images were converted to gray scale for quantification using Image J software as previously described^(^ ^9^ ^)^ (see Supplementary Detailed Materials and Methods).

### Reverse transcription‐quantitative polymerase chain reaction

RNA was isolated from CRISPR‐edited hFOB1.19 cells after 7 days of differentiation (39.5°C). RNA was subsequently purified, converted into cDNA, and subjected to qPCR using gene‐specific primers (see SupplementaryTable S1). Results were exported, normalized to GAPDH, and fold‐change calculated using Cq (ΔR) values and the comparative CT method (ΔΔCT method).^(^ ^15^ ^)^ (see Supplementary Detailed Materials and Methods for details).

### Western blotting

Plates seeded with CRISPR‐edited hFOB1.19 cells were grown at both 33.5°C and 39.5°C for 7 days, then cells were collected. Cells were washed and lysed on ice. Supernatants containing whole‐cell lysates were collected, total protein concentration was measured, and samples were normalized. Whole‐cell lysate was loaded onto gels then transblotted onto membrane stacks. Membranes were blocked in nonfat dry milk and incubated overnight at 4°C with EPDR1 antibody (ab197932; Abcam) and α‐tubulin antibody (sc‐58666; Santa Cruz Biotechnology). On the following day, membranes were washed, incubated with horseradish‐peroxidase–linked secondary antibodies (Santa Cruz Biotechnology), washed again, developed with chemiluminescent substrate, and visualized using the iBright FL1500 Imaging System (Thermo). Quantification of Western immunoblotting bands was performed using the build‐in software on the iBright system (see Supplementary Detailed Materials and Methods).

## Results

### RNAi knock‐down of *EPDR1* gene expression and alkaline phosphatase activity in differentiated hFOB1.19 cells

To validate differentiated hFOB1.19 cells as a comparable model to human mesenchymal stem cell (hMSC)‐derived osteoblasts for our line of investigation, we carried out RNAi targeting of *EPDR1* expression in hFOB1.19 cells. Our previous RNAi targeting of *EPDR1* in hMSC‐derived osteoblasts resulted in lower levels of both ALP staining and ARS staining.^(^ ^9^ ^)^ ALP activity is necessary for hydroxyapatite deposition and mineralization,^(^ ^16^ ^)^ whereas ARS stains for the deposition of calcium.^(^ ^17^ ^)^ However, though hFOB1.19 cells undergo osteoblast differentiation, they do not produce an appreciable amount of calcium for detection by ARS.^(^ ^18^ ^)^ Therefore, we elected to use ALP staining alone for assessment of differentiation. To confirm the same response as observed in hMSC‐derived osteoblasts, we carried out RNAi targeting of *EPDR1* expression in differentiated hFOB1.19 cells. ALP staining was markedly increased during differentiation of hFOB1.19 cells, while it was absent in undifferentiated cells and in *EPDR1* RNAi‐treated cells under differentiating conditions. Indeed, this reduction was more pronounced than our observations in hMSC‐derived osteoblasts (Supplementary Fig. S3 *A*,*B*).^(^ ^9^ ^)^ This confirmed that the hFOB1.19 cell line is a suitable cellular model to study preliminary osteoblast differentiation, and that the *EPDR1* gene product plays a functional role during differentiation of both hMSC‐derived osteoblasts and hFOB1.19 cells.

### Lentiviral CRISPR‐Cas9 targeting in hFOB1.19 cells of the putative enhancer region harboring key ‘*STARD3NL*’ proxy SNPs

We elected to employ a lentiviral‐CRISPR‐Cas9 construct containing mCherry to target the putative enhancer harboring the three proxy SNPs in strong LD with sentinel SNP rs6959212 at the ‘*STARD3NL*’ locus, which are all in close proximity (rs1524068, rs6975644, rs940347).^(^ ^9^ ^)^ Given that the three proxy SNPs are harbored within a 204‐bp region and indistinguishable from each other with respect to LD, we designed a pooled set of three sgRNA binding sites flanking each side of the proxy SNP set to delete the entire region of open chromatin (see Supplementary Fig. S2). mCherry expression in CRISPR targeted cells was used to visualize lentiviral transduction efficiency and averaged approximately 87% (see Supplementary Figs. S1 and S4). This high efficiency allowed us to proceed without cell sorting; however, it should be noted that as a result of this approach, all subsequent results included at least 10% of nontargeted cells.

### PCR and multiplexed sequencing validation of pooled CRISPR hFOB1.19 cells

**Fig. 2:**
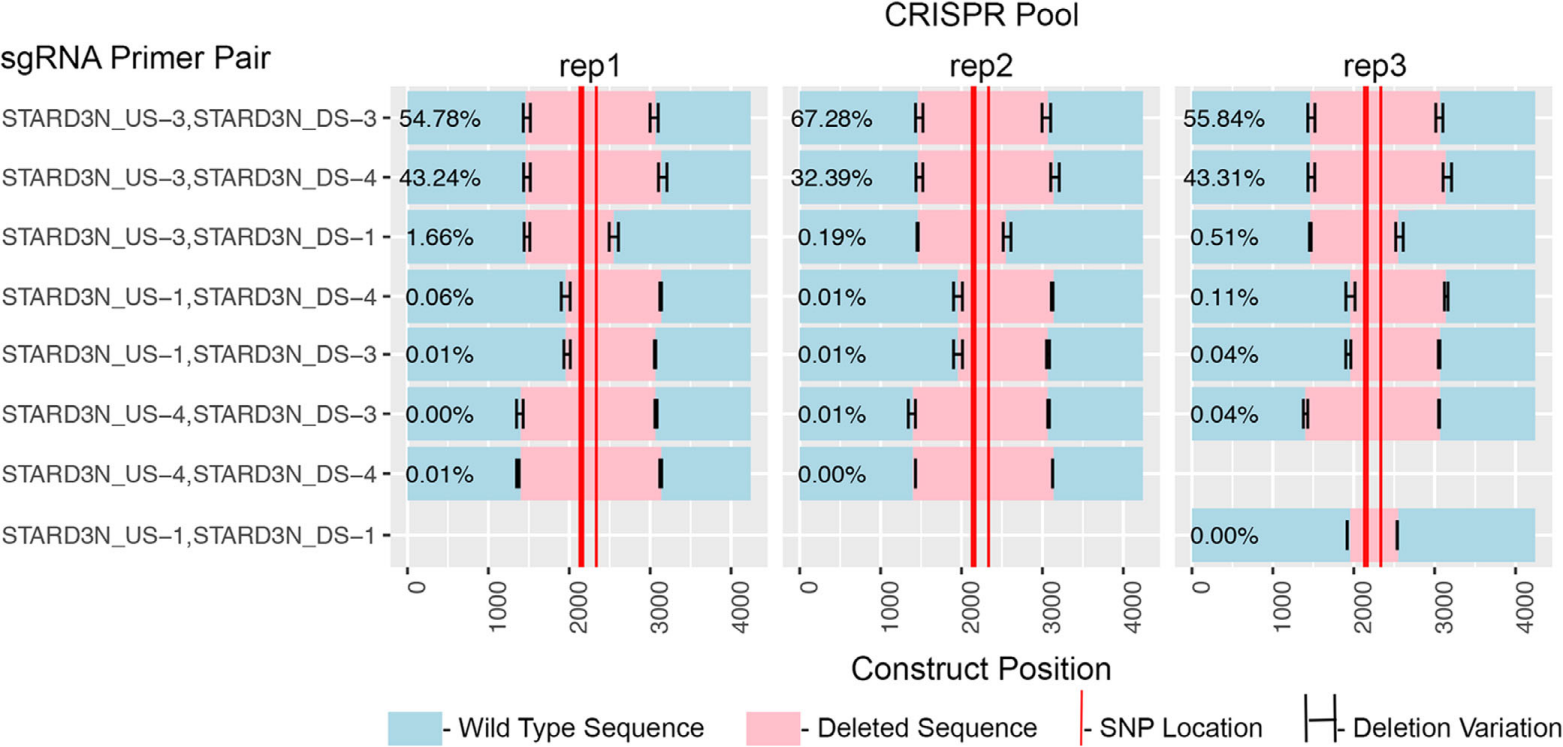
Multiplexed sequencing of clustered regularly interspaced short‐palindromic repeat (CRISPR)‐edited hFOB1.19 cell PCR products confirms proxy SNP deletions and reveals the efficient single‐guide RNA (sgRNA) pairs. Three biological replicates are shown. Teal indicates WT sequence, pink indicates CRISPR‐cas9 deleted regions, and the red bars indicate proxy‐SNP locations. The upstream‐3/downstream‐3 (US‐3/DS‐3) sgRNA combination produced the highest percentage of deletions (average 59.3%) followed by the US‐3/DS‐4 combination (average 39.6%). Variability in the CRISPR cut sites is indicated by the horizonal error bars.

PCR primers flanking the targeted region were used to amplify genomic DNA derived from the CRISPR‐edited hFOB1.19 cells (see Supplementary Fig. S2 and Supplementary Table S1). These PCR products were further selected for specificity with a nested set of PCR primers. PCR products from both WT and CRISPR deletions fell within the predicted size range (Supplementary Fig. S5). The WT product was 2370 bp in size, whereas deletions in the CRISPR‐edited pooled cells ranged from 595 to 1739 bp (PCR band size 631–1775 bp). To further verify each CRISPR deletion, all PCR products were sequenced using a multiplexed next‐generation sequencing adapter primer strategy modified from previously published studies^(^ ^19^, ^20^ ^)^ (see Supplementary Fig. S2). Although there is some variability among replicates, it is apparent that the upstream (US‐3), downstream (DS‐3) sgRNA pair were the most efficient at CRISPR‐Cas9–mediated deletion of the proxy SNP region, followed closely by the US‐3 and DS‐4 pair (Fig. 2). These sgRNA combinations correspond to bands in Supplementary Fig. S5. Other sgRNA pairs‐generated CRISPR‐Cas9 deletions to varying degrees in each replicate of CRISPR pool cells. Based on this validation of the deletion of the region harboring the proxy SNPs, we proceeded with functional analysis of the pooled CRISPR‐edited hFOB1.19 cells.

### qPCR analysis of *EPDR1* RNA expression in pooled CRISPR hFOB1.19 cells

**Fig. 3:**
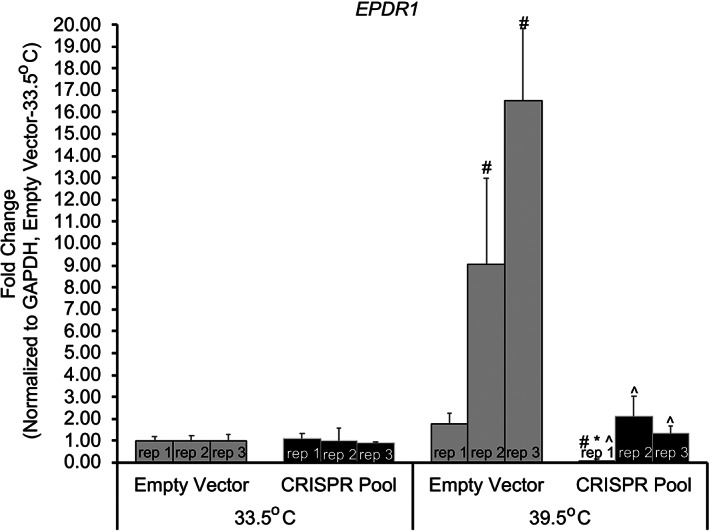
RT‐qPCR of clustered regularly interspaced short‐palindromic repeat (CRISPR)‐edited hFOB1.19‐derived RNA reveals *EPDR1* mRNA levels increase when normalized to GAPDH expression and permissive control. Three biological replicates are shown for each condition. All samples are normalized to control (empty vector) at permissive (33.5°C) growth. This normalization reveals a large increase (up to 16‐fold) in *EPDR1* mRNA levels when hFOB1.19 cells differentiate (39.5°C) but levels return to basal levels when the proxy‐SNPs contain region is deleted by CRISPR‐cas9. *p* Values (*t* test): # = empty vector‐33.5°C versus all, * = CRISPR pool‐33.5°C versus CRISPR pool‐39.5°C, ^ = empty vector‐39.5°C versus CRISPR pool‐39.5°C, all marked samples have a *p* < 0.04.

Given that distal cis‐regulatory elements generally regulate gene expression via contact to physically cooperate with the promoter and/or proximal US regulatory regions, we hypothesized that contacts between the putative causal SNP region and the *EPDR1* promoter regulate *EPDR1* gene expression. To test this, we measured *EPDR1* expression by RT‐qPCR using gene‐specific primers (see Supplementary Table S1) on control (empty vector, ie, the LentiCRISPRv2‐mCherry construct containing no sgRNA sequence) or CRISPR‐targeted hFOB1.19 cells grown for 5 days under either permissive or differentiation conditions. When all samples were first normalized to *GAPDH* expression and then normalized to the permissive empty‐vector control samples, a large increase in *EPDR1* mRNA was observed in the differentiated control samples, but *EPDR1* mRNA levels remained significantly lower in the differentiated pooled CRISPR‐edited samples (Fig. 3). No difference in *EPDR1* mRNA expression was observed in the cells growing under undifferentiated conditions (Fig. 3). Normalization of CRISPR samples to *GAPDH* for the corresponding permissive or differentiated controls, respectively, revealed levels of *EPDR1* mRNA in differentiated CRISPR‐edited hFOB1.19 cells were reduced three‐ to sevenfold compared with controls and unchanged in the undifferentiated cells (Supplementary Fig. S6). Taken together, these data strongly support a role for the direct physical contact between the putative enhancer region harboring the key proxy SNPs and the *EPDR1* gene during osteoblast differentiation.

### Western blot analysis of EPDR1 protein expression in pooled CRISPR‐edited hFOB1.19 cells

**Fig. 4:**
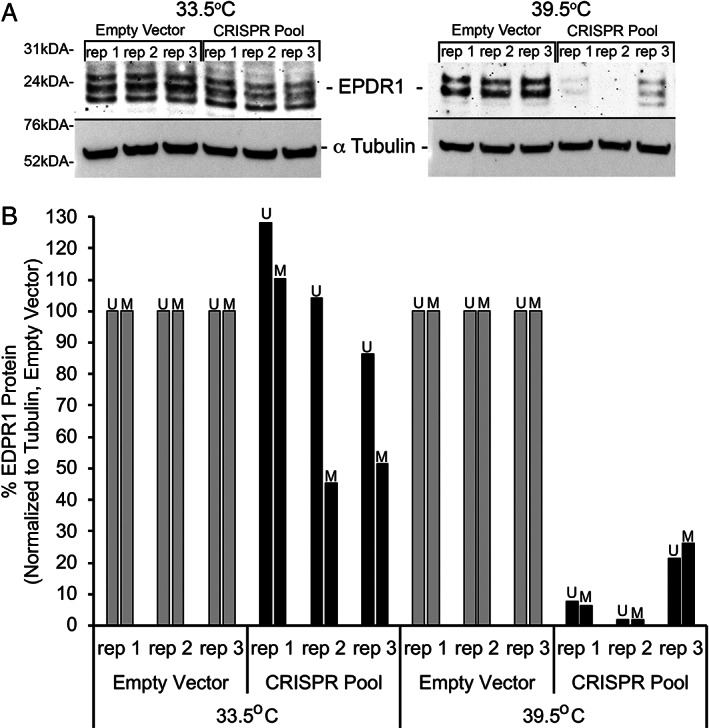
Western Immunoblotting reveals a decrease in EPDR1 protein levels specific to clustered regularly interspaced short‐palindromic repeat (CRISPR)‐edited hFOB1.19 cells differentiated for 7 days. Three biological replicates are shown for each condition. (*A*) Western immunoblotting detected bands for all three isotypes of EPDR1 (25 kDa) with a decrease in EPDR1 band intensities in differentiated CRISPR‐edited hFOB1.19 samples. Equal loading was verified with the housekeeping α‐tubulin antibody (55 kDa). (*B*) Quantification of the upper band (U) and middle band (M) that are present across all samples shows a decrease of approximately 90% on average for both U and M bands when compared with controls (empty vector).

To confirm that lower expression levels of *EPDR1* mRNA translate to lower levels of EPDR1 protein in our pooled CRISPR‐edited hFOB1.19 cells, we grew each replicate under both permissive and differentiation conditions for 7 days and subjected whole‐cell lysates to immunoblot analysis. As shown in Fig. 4*A*, EPDR1 protein‐expression levels were markedly reduced in the pooled CRISPR‐edited hFOB1.19 cells grown under differentiation conditions, but not in those grown under permissive conditions, which is consistent with the results observed for *EPDR1* mRNA levels. Quantification of both the upper band (U) and middle band (M) present across all samples showed approximately a 70% to 90% decrease in EPDR1 protein levels when normalized to tubulin, but in the differentiated samples only (Fig. 4*B*).

### Alkaline phosphatase activity in pooled CRISPR‐edited hFOB1.19 cells

**Fig. 5:**
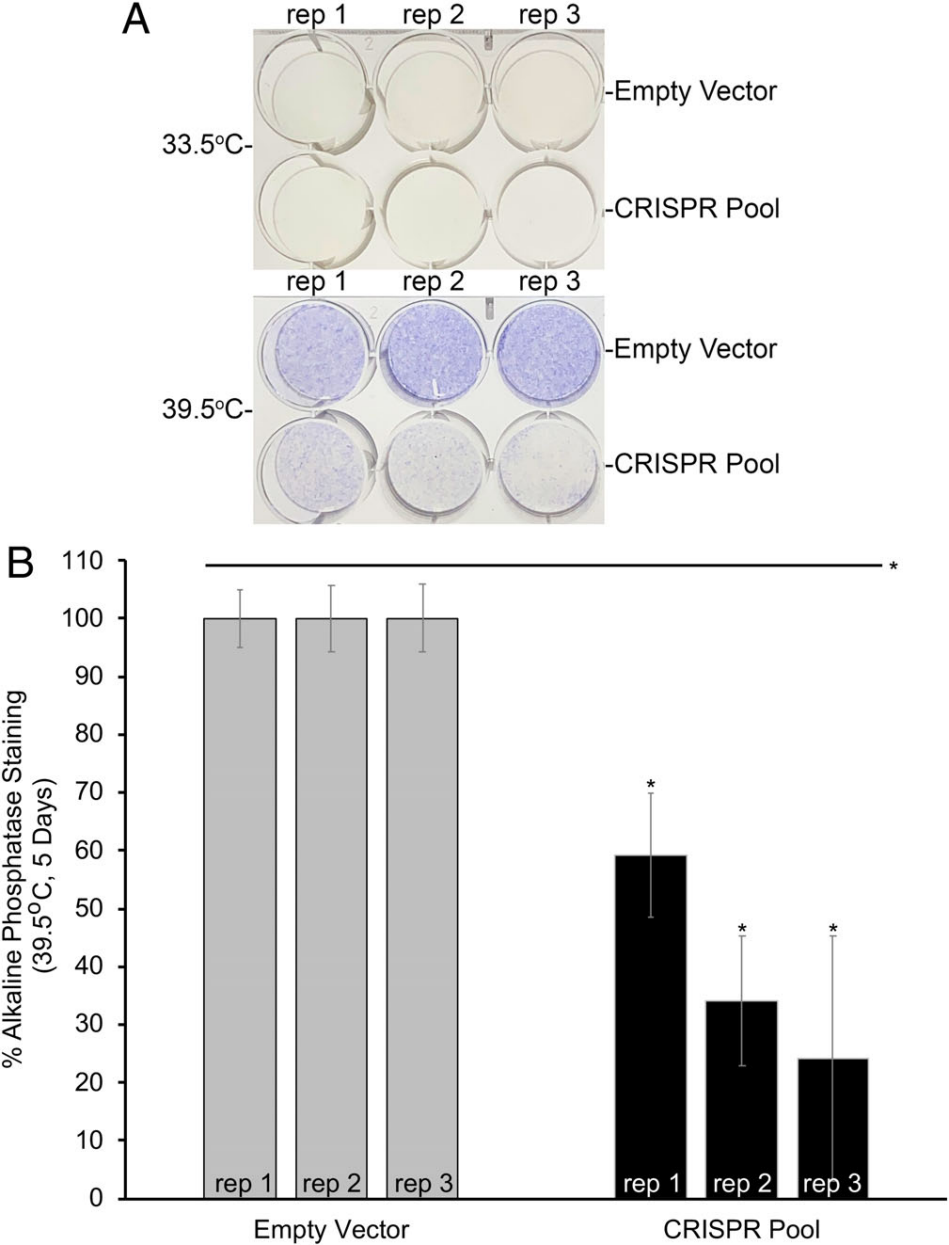
Alkaline phosphatase staining (ALP) is reduced in clustered regularly interspaced short‐palindromic repeat (CRISPR)‐edited hFOB1.19 cells differentiated for 5 days. Three biological replicates are shown for each condition. (*A*) ALP staining of plates grown under both permissive (33.5°C) and differentiation (39.5°C) conditions. Purple color indicates ALP activity. (*B*) Quantification of ALP staining shows an average 61% decrease in ALP staining in differentiated hFOB1.19 samples. *p* Values (*t* test): * = empty vector‐39.5°C versus CRISPR pool‐39.5°C, all marked samples have a *p* < 0.0005.

ALP activity is an important biomarker of osteoblast differentiation; therefore, we sought to confirm that this decrease in *EPDR1* expression leads to functional differences in ALP activity in differentiated hFOB1.19 cells lacking the putative noncoding causal SNP region. Pooled CRISPR‐edited hFOB1.19 replicates were generated under permissive and differentiation conditions for 5 days. Plates were then assessed for ALP activity in the same manner used for RNAi‐treated cells. As shown in Fig. 5*A*, differentiation‐induced ALP staining was reduced in CRISPR‐edited hFOB1.19 cells compared with controls, and quantification of ALP staining revealed a 40% to 76% decrease in this staining (Fig. 5*B*). We presume a portion of the staining in the CRISPR‐edited cells is based on the presence of WT cells (~10%) in our pooled replicates. This decrease in ALP staining in differentiated CRISPR‐edited hFOB1.19 cells further implicates *EPDR1* in the differentiation of osteoblasts. These results also validate the importance of the candidate causal SNP region and its regulatory function on the putative effector *EPDR1* gene promoter.

## Discussion

In our previous study, we implicated a putative regulatory region harboring a tight cluster of three BMD GWAS proxy SNPs interacting with *EPDR1*, a gene not previously known to be involved in bone metabolism.^(^ ^9^ ^)^ It should be noted that the previous study identified other novel genes implicated in osteoblastogenesis using this method; the same techniques could be more generally applied to other GWASs and cell model systems. In this current study, we sought to fully validate the regulatory connection between the SNP harboring region at the ‘*STARD3NL*’ locus and the implicated *EPDR1* gene. Deleting this region coinciding with the putative underlying causal SNP in hFOB1.19 cells by CRISPR‐Cas9 provided direct evidence for an *EPDR1* regulatory function of this region at both the RNA and protein levels, and interestingly, this regulation is cell differentiation state‐specific. The nearby gene *SFRP4* (see Fig. 1) was not detectable by qPCR and showed no significant change on Western blots in hFOB1.19 cells (data not shown). Thus, these studies also support our prior initial evidence that *EPDR1* plays a role in osteoblastogenesis by validating the putative regulatory region has an effect on ALP activity through CRISPR‐based perturbation.

GWASs have proven very informative in identifying highly associated regions of interest within the genome, but very often the identified SNP is not casual and the actual effector gene still has to be elucidated. In addition, target validation follow‐up studies are needed to fully verify the observed interactions and their regulatory consequences. In this case, multiple BMD GWASs reported an intergenic regulatory region at the ‘*STARD3NL*’ locus; hence, we subsequently implicated *EPDR1* as a putative target effector gene. This follow‐up validation study was able to confirm both a regulatory role for this proxy SNP region in *EPDR1* expression and further supported a role for EPDR1 in osteoblastogenesis.

EPDR1 is a relatively novel protein whose function is still being resolved. It may serve as a type II transmembrane protein,^(^ ^21^ ^)^ but other studies have suggested it is secreted and that it could play a direct transcriptional regulatory role.^(^ ^22^ ^)^ EPDR1 is also known to interact with extracellular matrix proteins that are known to play a role in osteoblast differentiation.^(^ ^21^, ^22^, ^23^ ^)^ Furthermore, EPDR1 peptides have been shown to play a role in transcription via AP‐1,^(^ ^23^ ^)^ a TF involved in osteoblast differentiation through TGF‐β, PTH, and 1,25‐dihydroxy vitamin D^(^ ^24^, ^25^ ^)^; thus, AP‐1 transcriptional activity may reveal another role for *EPDR1* in this setting, and therefore warrants follow‐up efforts. These other studies suggest that *EPDR1* represents an attractive novel target for bone metabolism. Although EPDR1 downregulation impairs gene expression, elucidating the impact of reduced EPDR1 on matrix composition at the protein level will be essential.

Although the present study confirms a regulatory role for the proxy SNP‐harboring open region in *EPDR1* gene regulation because of the tight proximity of the three BMD GWASs' implicated proxy SNPs, the identity of the exact causal variant remains unknown. Identification of the precise SNP(s) involved will require more precise techniques than CRISPR‐Cas9– mediate deletion of this region. Other sequence‐specific techniques like CRISPR inhibition (CRISPRi) and/or CRISPR activation (CRISPRa), which have no endonuclease activity but either inhibit or activate at the sgRNA guide position without modifying the genome or CRISPR‐Cas9‐based synchronous programmable adenine and cytosine editor (SPACE),^(^ ^26^ ^)^ may allow more precise targeting of the individual alleles. Potential binding sites for TFs NFκB‐cRel and AP‐2A are present at rs1524068, as well as ARP‐1 at rs940347. NFκB‐cRel is known to be an osteoblast‐related TF^(^ ^24^ ^)^ and has been shown to be a crucial factor responsible for impaired bone formation in osteoporosis,^(^ ^25^ ^)^ whereas AP‐2A has been shown to enhance the osteogenic differentiation potential of MSCs.^(^ ^27^ ^)^ Thus, further studies using electrophoretic mobility shift assay (EMSA) and/or chromatin immunoprecipitation (ChIP) have the potential to verify TF binding at these SNPs and thus implicate potential roles for these TFs in *EPDR1* gene regulation.

This work also builds on efforts by other groups to connect osteoporosis‐reported GWAS signals directly to the transcription of a distal gene, and thus DS osteoblastogenesis.^(^ ^28^ ^)^ Although additional studies are needed to further explore the regulation of *EPDR1* via the *‘STARD3NL’* locus and to understand its mechanistic function, our cumulative data clearly point to *EPDR1* being involved in bone differentiation processes and thus represents a new target for BMD‐related osteoporosis studies.

## Conflicts of interest

The authors report no conflicts of interest.

### Peer Review

The peer review history for this article is available at https://publons.com/publon/10.1002/jbm4.10531.

## Supporting information

**Fig. S1.** Bright field and Texas red fluorescent microscopy of CRISPR‐edited hFOB1.19 cells at 10X magnification. Comparison of the same field under both bright field and Texas Red fluorescence shows a high number of mCherry positive cells in both empty vector and CRISPR pool cells for all three biological replicates. Scale bar = 200 μM.

**Fig. S2.** CRISPR‐cas9 primer design for the ‘*STARD3NL*’ locus proxy SNP region. Proxy SNPs (rs940347, rs6975644, and rs1524068) are indicated at the center of the CRISPR region, sgRNAs are indicated by boxes, PCR primers are indicated by arrows and multiplexed sequencing primers are indicated by tailed‐arrows. Diagram is to scale with regard to primer locations.

**Fig. S3.** RNAi targeting of *EPDR1* expression decreases alkaline phosphatase staining (ALP) in differentiated hFOB1.19 cells. (*A*) Alkaline phosphatase staining (ALP) produces purple staining upon activation of alkaline phosphatase during differentiation (39.5°C) but no staining is visible during permissive growth (33.5°C). *EPDR1* RNAi decreases ALP staining after 5 days of differentiation (39.5°C). (*B*) Quantification shows a doubling of ALP staining during differentiation in control RNAi samples, but levels return to near baseline when treated with *EPDR1* RNAi. *P*‐values (t‐test): # = Control RNAi‐33.5°C vs. All Groups, * = Control RNAi‐39.5°C vs. *EPDR1* RNAi‐39.5°C, all marked samples have a *p* < 0.004.

**Fig. S4.** mCherry positive cell counting shows high lentiviral transduction efficiency. Trypsinized hFOB1.19 cells transduced with either empty vector (gray) or CRISPR pool (black) lentivirus were counted using both bight field and Texas Red fluorescence. The ratio between the two was used to calculate transduction efficiency and averaged ~87% among the three biological replicates.

**Fig. S5.** PCR products generated across the ‘*STARD3NL*’ (sentinel rs6959212) locus proxy SNPs region (rs1524068, rs6975644, rs940347) from genomic DNA reveal a variety of deletions in CRISPR‐edited hFOB1.19 cells. The wild type PCR product band size is 2370 bp, the smallest deletion (595 bp) generates a PCR product band size of 1775 bp and the largest deletion (1739 bp) generates a PCR product band size of 631 bp. Other sgRNA combinations generate PCR products within the CRISPR deletion range in each of the three biological replicates.

**Fig. S6.** RT‐qPCR of CRISPR‐edited hFOB1.19 derived RNA reveals no change in *EPDR1* mRNA expression levels in cells grown under permissive (33.5°C) conditions but a dramatic decrease in *EPDR1* mRNA expression levels in CRISPR‐edited cells when differentiated (39.5°C) for 7 days. All three biological replicates were normalized to GAPDH then Empty Vector. pValues (t‐test): # = Empty Vector vs CRISPR pool, * = 33.5°C vs 39.5°C, ^ = Empty Vector‐39.5°C vs CRISPR‐39.5°C, all marked samples have a *p* < 0.04.

**Table S1** Table contains the sequence of all primers used in these studies including: RNAi, sgRNAs, PCR primers, multiplexed sequencing primers and RT‐qPCR primers.

**Appendix S1** Detailed Materials and Methods. Supplementary material and methods containing reagent and equipment details along with protocol level experimental details.
