# Supplementary figures and images for: CRISPR‐Cas9–Mediated Genome Editing Confirms EPDR1 as an Effector Gene at the BMD GWAS‐Implicated ‘STARD3NL’ Locus
Source: JBMR Plus. 2021 Jul 23;5(9):e10531. doi: 10.1002/jbm4.10531 (PMC8441377; doi:10.1002/jbm4.10531)

# Supplemental Figure 1

## Empty Vector

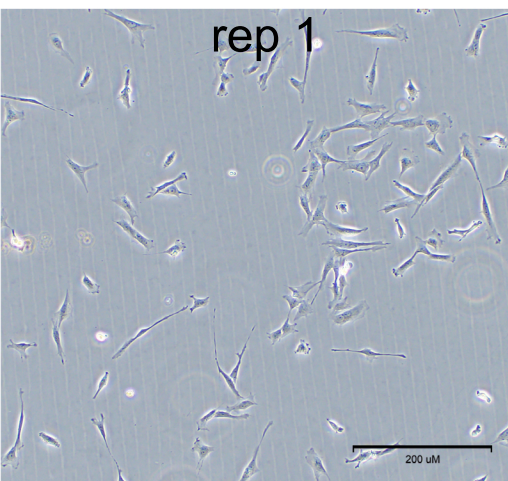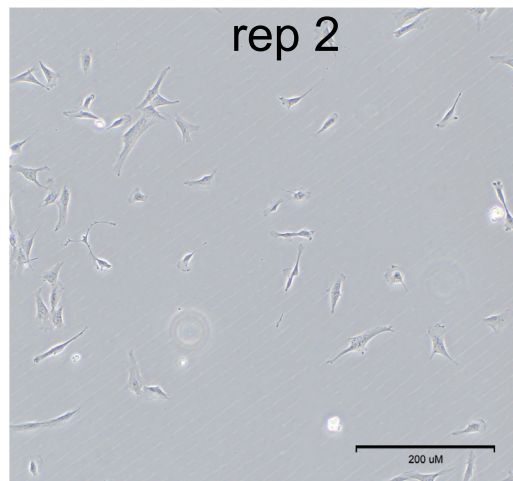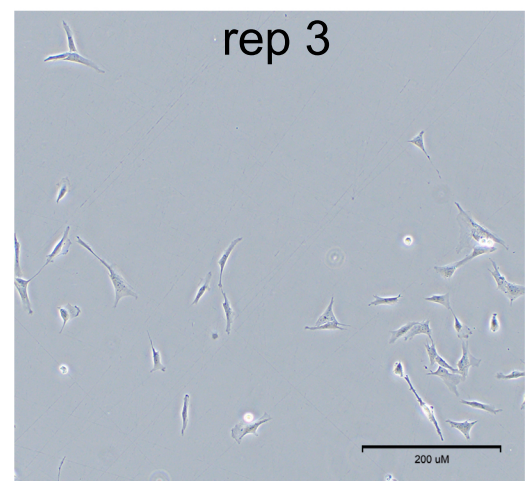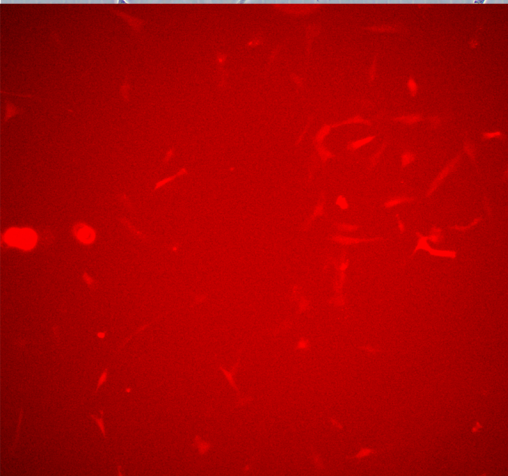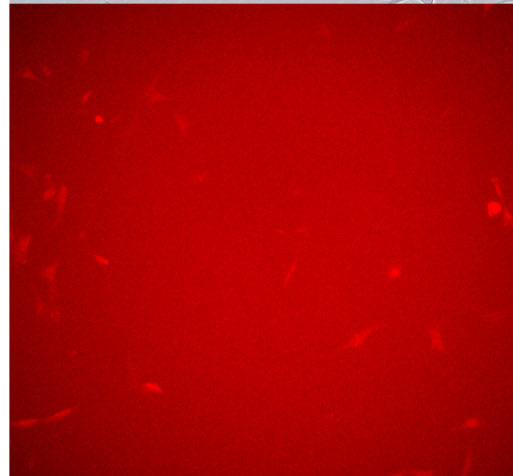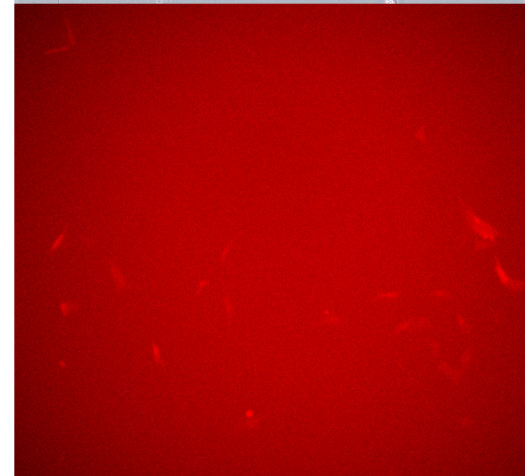

## CRISPR Pool

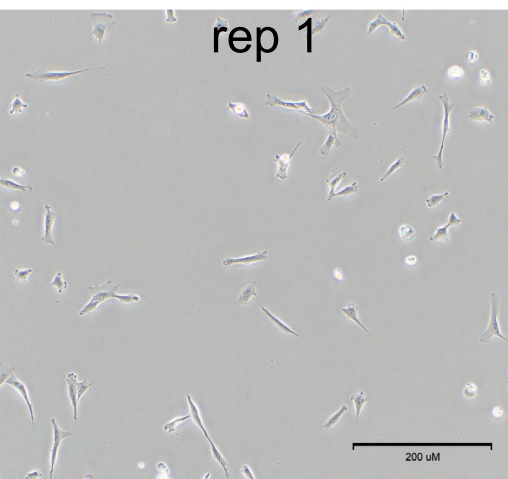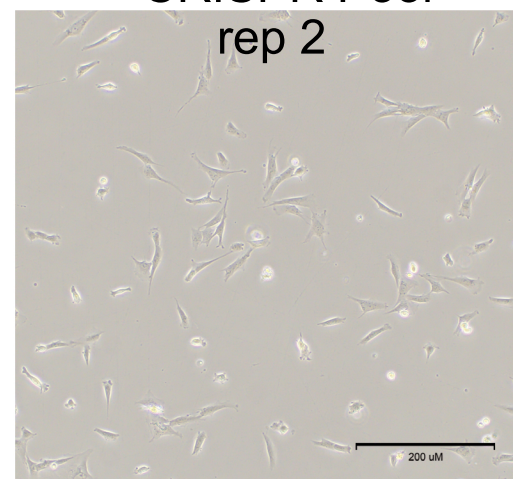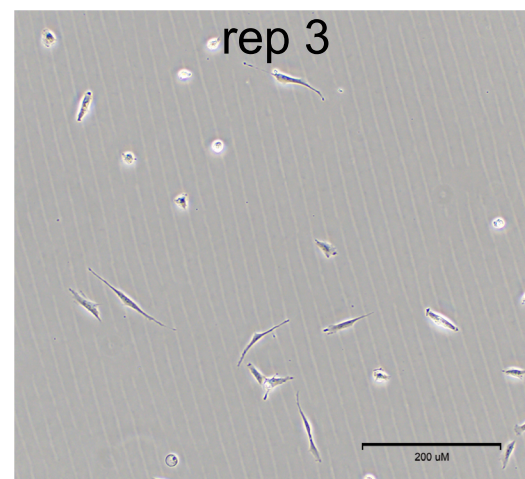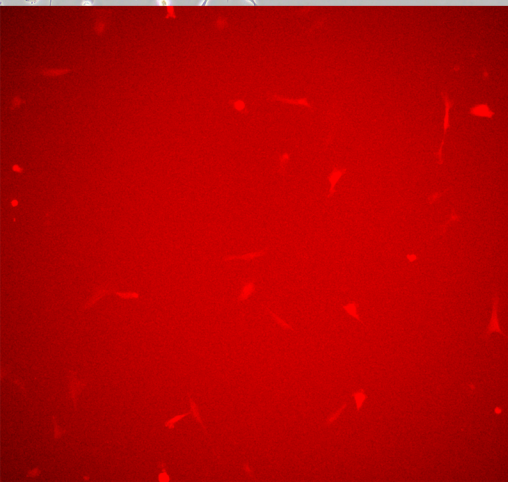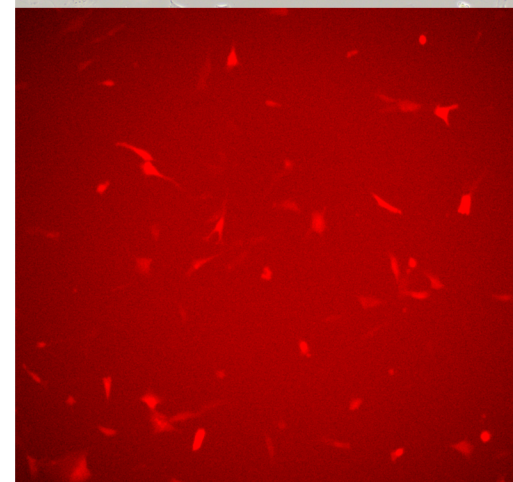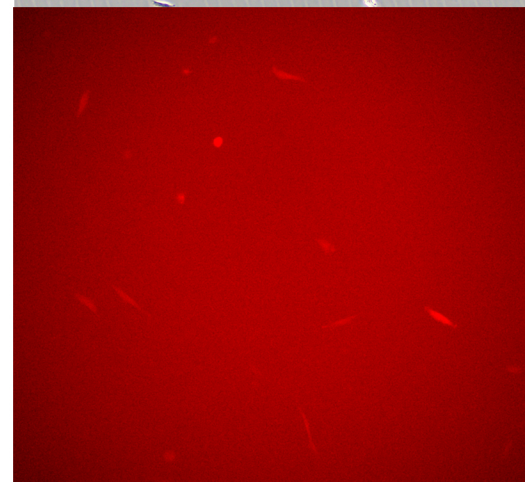

Supplement: Supplementary file 1 — Fig. S1. Bright field and Texas red fluorescent microscopy of CRISPR‐edited hFOB1.19 cells at 10X magnification. Comparison of the same field under both bright field and Texas Red fluorescence shows a high number of mCherry positive cells in both empty vector and CRISPR pool cells for all three biological replicates. Scale bar = 200 μM. [file JBM4-5-e10531-s002.pdf]

# Supplemental Figure 2

## CRISPR Design at '*STARD3NL*' Locus (Sentinel rs6959212) Proxy SNPs

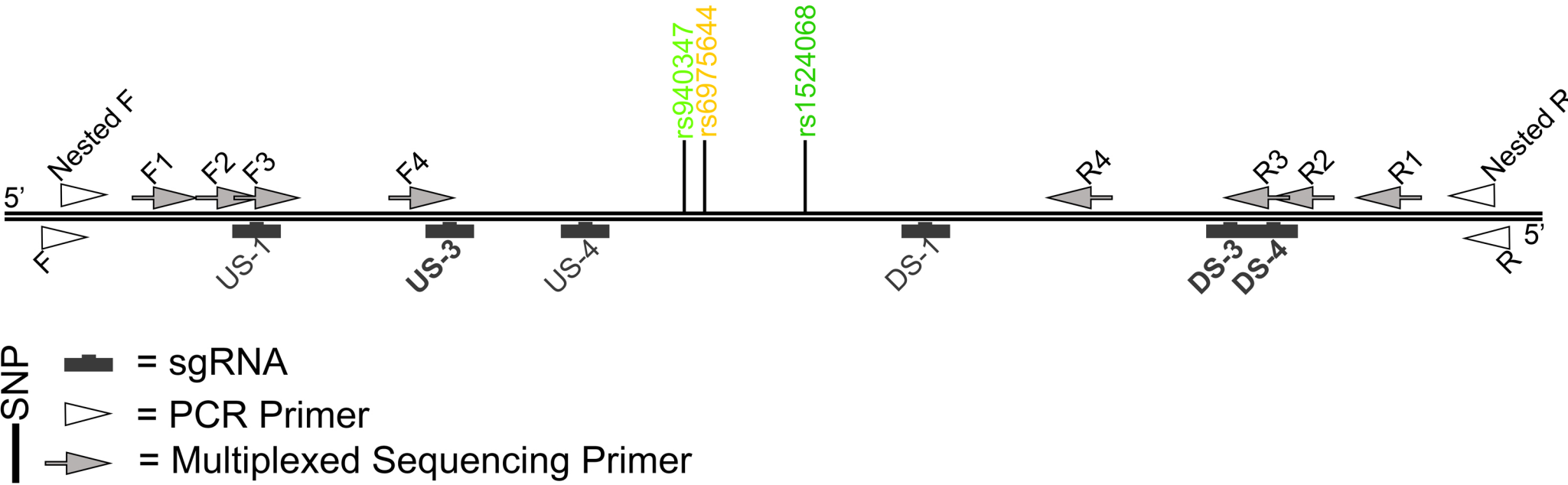

Supplement: Supplementary file 2 — Fig. S2. CRISPR‐cas9 primer design for the ‘STARD3NL’ locus proxy SNP region. Proxy SNPs (rs940347, rs6975644, and rs1524068) are indicated at the center of the CRISPR region, sgRNAs are indicated by boxes, PCR primers are indicated by arrows and multiplexed sequencing primers are indicated by tailed‐arrows. Diagram is to scale with regard to primer locations. [file JBM4-5-e10531-s001.pdf]

# Supplemental Figure 3

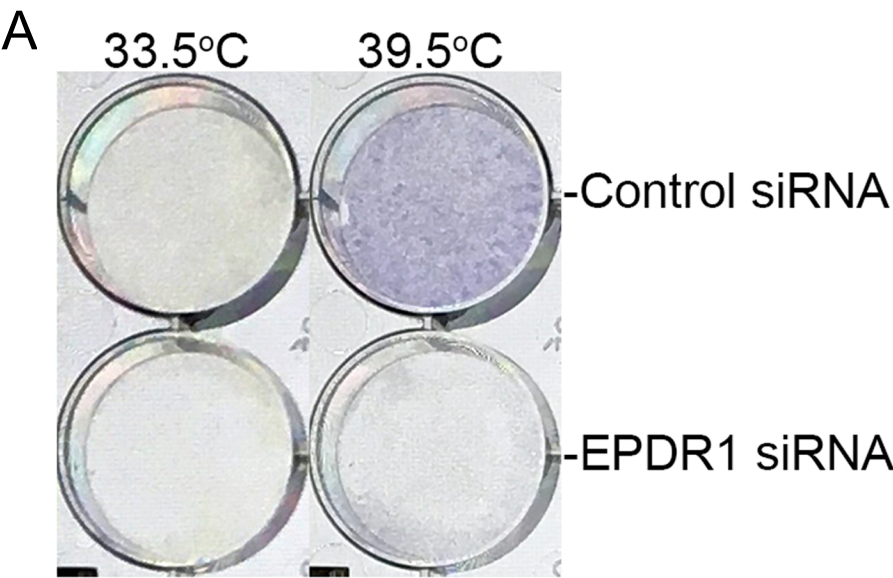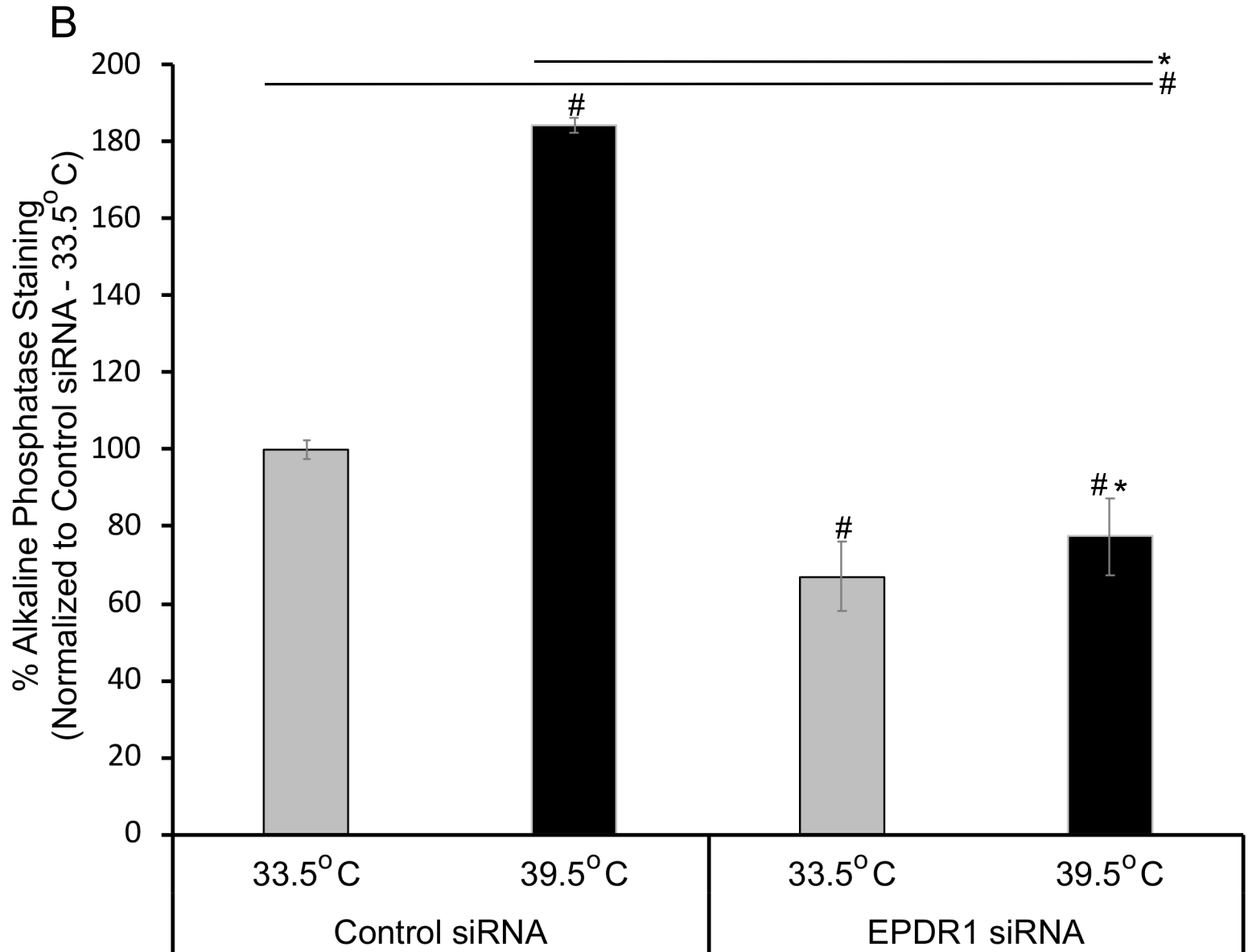

Supplement: Supplementary file 3 — Fig. S3. RNAi targeting of EPDR1 expression decreases alkaline phosphatase staining (ALP) in differentiated hFOB1.19 cells. (A) Alkaline phosphatase staining (ALP) produces purple staining upon activation of alkaline phosphatase during differentiation (39.5°C) but no staining is visible during permissive growth (33.5°C). EPDR1 RNAi decreases ALP staining after 5 days of differentiation (39.5°C). (B) Quantification shows a doubling of ALP staining during differentiation in control RNAi samples, but levels return to near baseline when treated with EPDR1 RNAi. P‐values (t‐test): # = Control RNAi‐33.5°C vs. All Groups, * = Control RNAi‐39.5°C vs. EPDR1 RNAi‐39.5°C, all marked samples have a p < 0.004. [file JBM4-5-e10531-s007.pdf]

Supplemental Figure 4

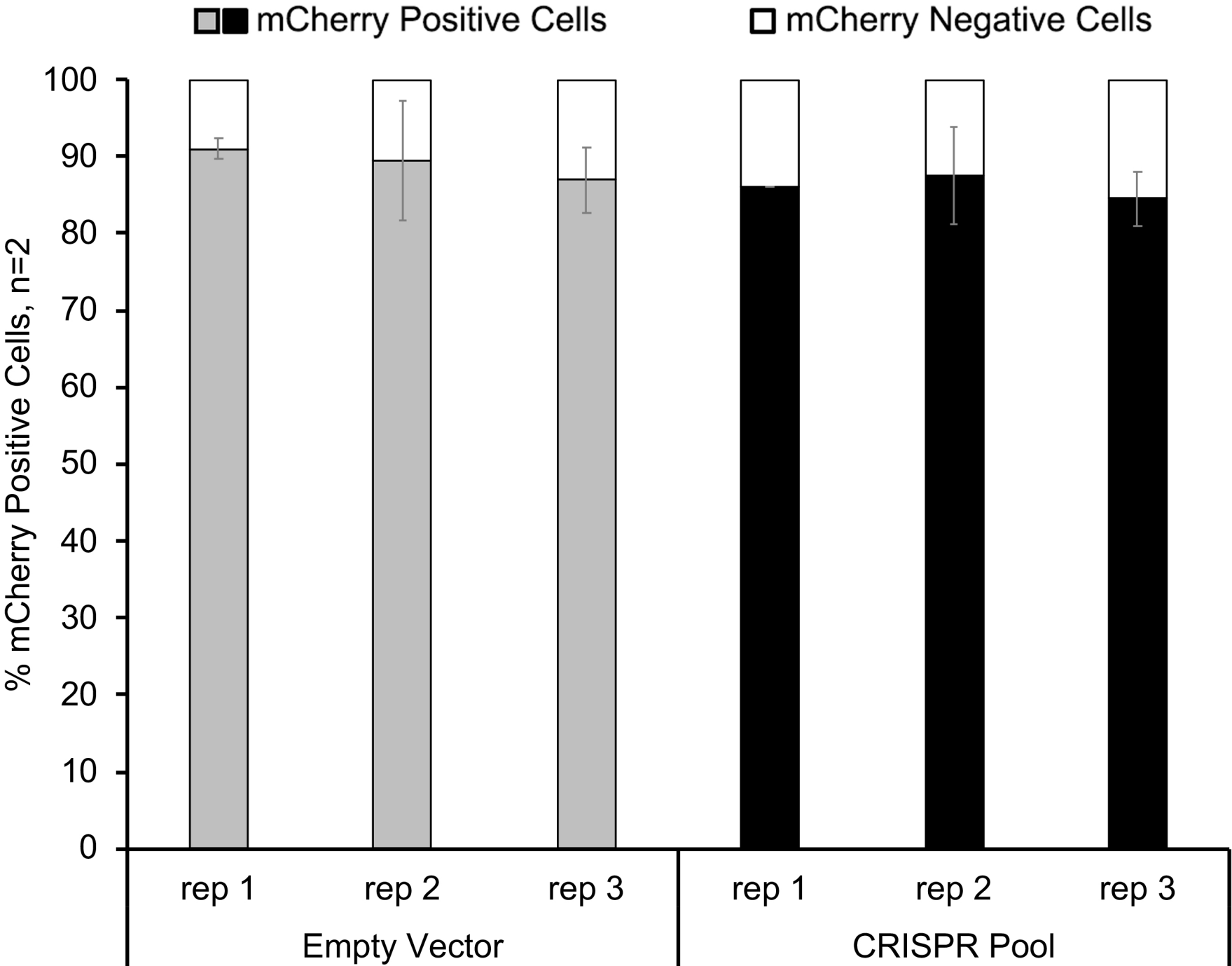

Supplement: Supplementary file 4 — Fig. S4. mCherry positive cell counting shows high lentiviral transduction efficiency. Trypsinized hFOB1.19 cells transduced with either empty vector (gray) or CRISPR pool (black) lentivirus were counted using both bight field and Texas Red fluorescence. The ratio between the two was used to calculate transduction efficiency and averaged ~87% among the three biological replicates. [file JBM4-5-e10531-s008.pdf]

# Supplemental Figure 5

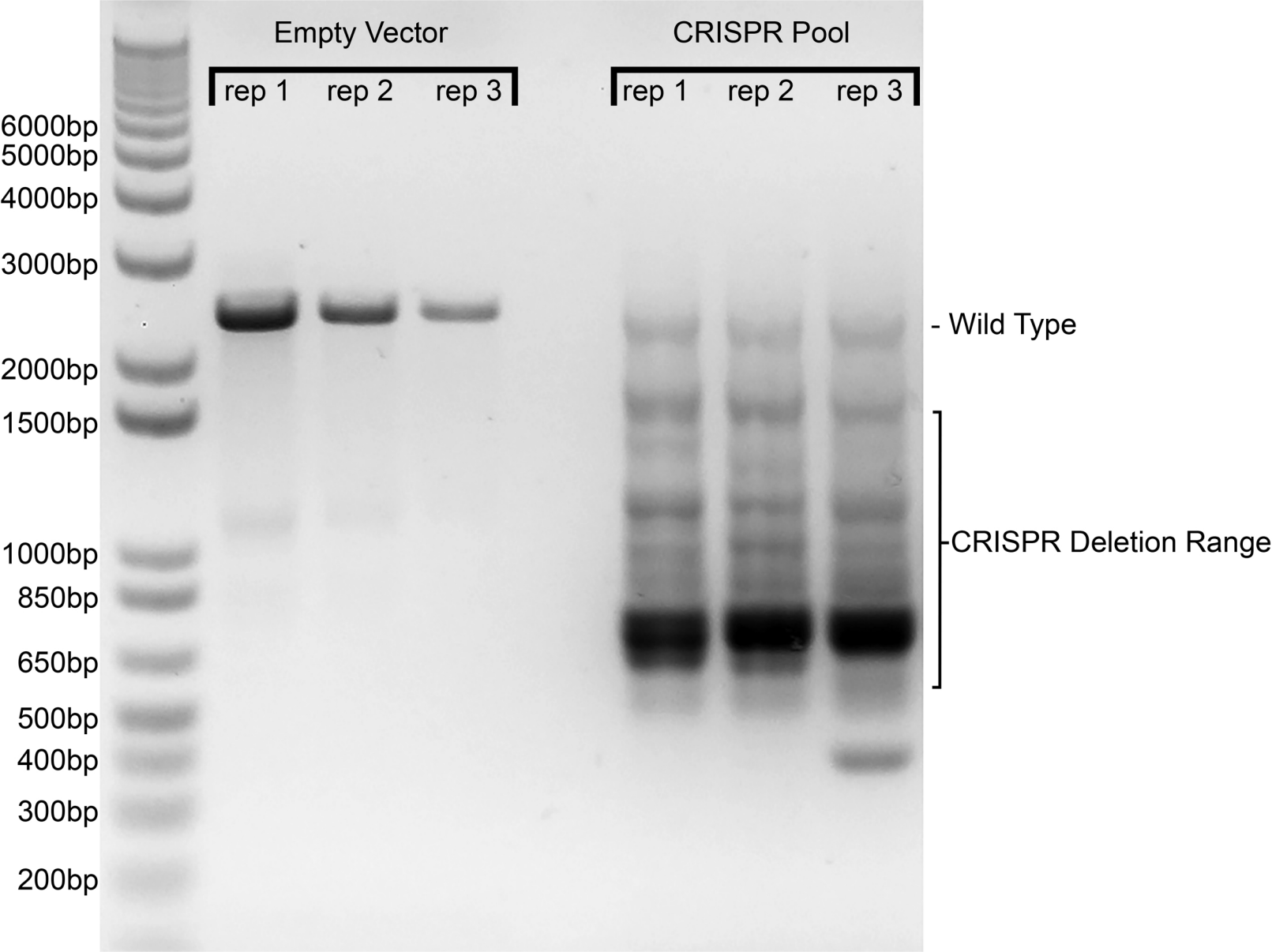

Supplement: Supplementary file 5 — Fig. S5. PCR products generated across the ‘STARD3NL’ (sentinel rs6959212) locus proxy SNPs region (rs1524068, rs6975644, rs940347) from genomic DNA reveal a variety of deletions in CRISPR‐edited hFOB1.19 cells. The wild type PCR product band size is 2370 bp, the smallest deletion (595 bp) generates a PCR product band size of 1775 bp and the largest deletion (1739 bp) generates a PCR product band size of 631 bp. Other sgRNA combinations generate PCR products within the CRISPR deletion range in each of the three biological replicates. [file JBM4-5-e10531-s003.pdf]

Supplemental Figure 6

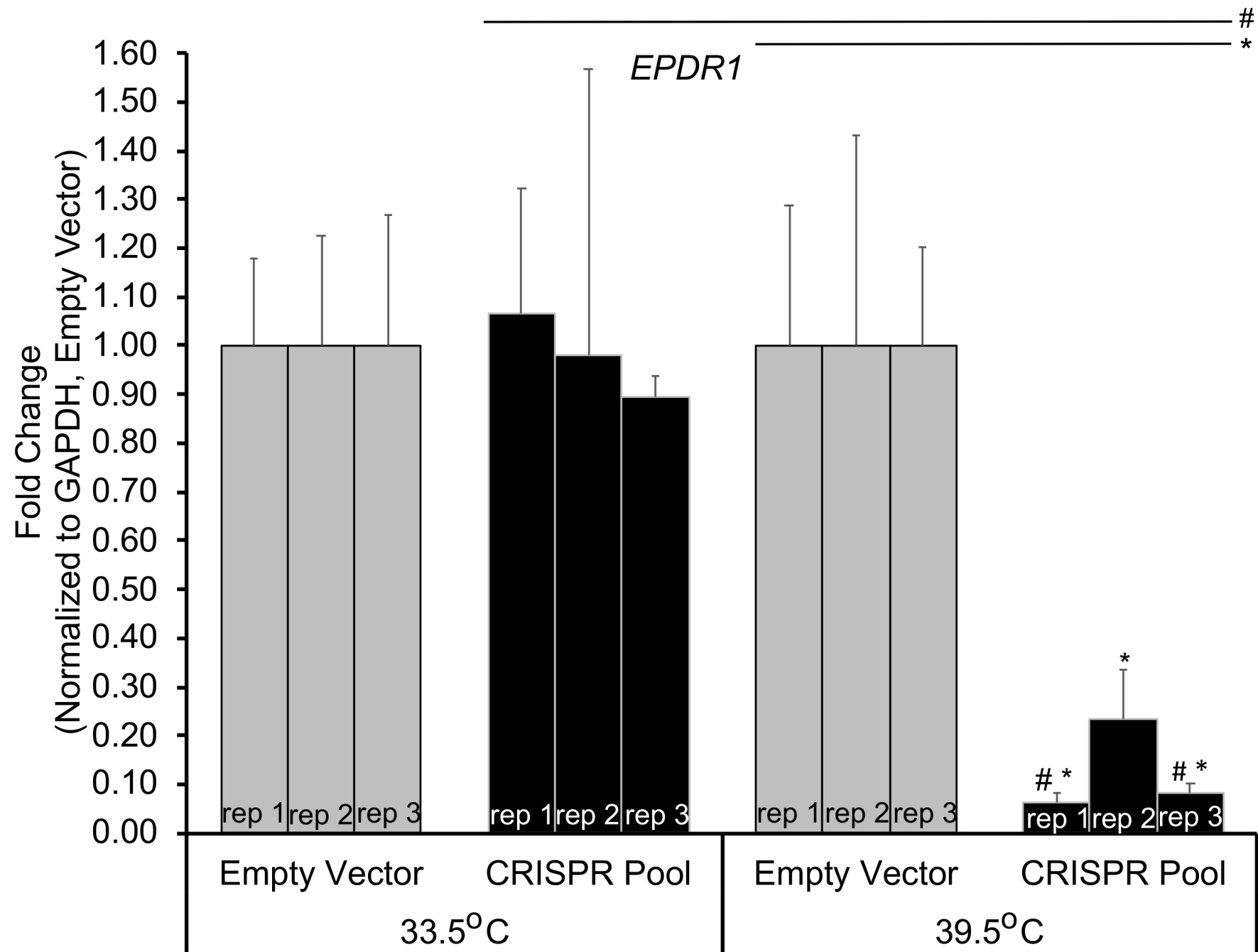

Supplement: Supplementary file 6 — Fig. S6. RT‐qPCR of CRISPR‐edited hFOB1.19 derived RNA reveals no change in EPDR1 mRNA expression levels in cells grown under permissive (33.5°C) conditions but a dramatic decrease in EPDR1 mRNA expression levels in CRISPR‐edited cells when differentiated (39.5°C) for 7 days. All three biological replicates were normalized to GAPDH then Empty Vector. pValues (t‐test): # = Empty Vector vs CRISPR pool, * = 33.5°C vs 39.5°C, ^ = Empty Vector‐39.5°C vs CRISPR‐39.5°C, all marked samples have a p < 0.04. [file JBM4-5-e10531-s006.pdf]
