# Supplementary material for: CRISPR‐Cas9–Mediated Genome Editing Confirms EPDR1 as an Effector Gene at the BMD GWAS‐Implicated ‘STARD3NL’ Locus
Source: JBMR Plus. 2021 Jul 23;5(9):e10531. doi: 10.1002/jbm4.10531 (PMC8441377; doi:10.1002/jbm4.10531)
Supplement: Supplementary file 8 — Appendix S1 Detailed Materials and Methods. Supplementary material and methods containing reagent and equipment details along with protocol level experimental details. [file JBM4-5-e10531-s005.docx]

**DETAILED MATERIALS AND METHODS**

**Cell Culture**

hFOB1.19 and 293T cells were purchased from ATCC (CRL-11372, CRL-­3216). hFOB1.19 cells were cultured in a 1:1 mixture of Ham's F12 Medium and Dulbecco's Modified Eagle's Medium containing no phenol red (Gibco, 21041025) and supplemented with 10% FBS and 0.3mg/mL G418 (Mediatech, MT30-234-CR). Cells were maintained using standard culture conditions at 33.5^o^C. Differentiation of cells into mature osteoblasts was accomplished by growing the cells at 39.5^o^C for 5-7 days for all experiments. 293T cells were cultured in DMEM Medium (Invitrogen, 11885084) supplemented with 10% FBS and 1X antibiotic/antimitotic (Invitrogen, 15240062) using standard culture conditions at 37^o^C.

**RNAi treatment**

Cells were seeded in 12-well plates at a density of 70K cells per well. The next day RNAi transfections were carried out using sets of 4 ON-TARGETplus RNAis (see **Supplemental, Table 1**) at 20nM final concentration and DharmaFECT1 Transfection Reagent (Dharmacon, T-2001-03) in Opti-MEM (Gibco, 31985062) according to manufacturer’s instructions. Twenty-four hours later, media was replaced with fresh growth media. Cells designated for differentiation into mature osteoblasts were moved to 39.5^o^C the following day and cells were allowed to grow until assayed for ALP staining after 5 days.

**CRISPR Constructs, Lentivirus Production, hFOB1.19 Infection**

The LentiCRISPRv2-mCherry construct was purchased from Addgene (Plasmid #99154) while sgRNAs were synthesized by IDT with BsmB1 restriction enzyme overhangs, duplexed, 5’ phosphorylated and ‘Lab Ready’ (see **Supplemental, Table 1**). This allowed cloning sgRNAs into the LentiCRISPRv2-mCherry construct using a modified Golden Gate assembly method ^(12)^ ^(13)^ ^(14)^. Proper insertion of the sgRNAs into the construct was confirmed using Sanger sequencing, and a pool containing an equal molar ratio of sgRNA constructs was generated for transfection.

One and one half million 293T cells were seeded in 100mm tissue culture (TC) plates and allowed to adhere for 24hrs. Cells were transfected with 24 micrograms total DNA including equal ratios of LentiCRISPRv2-mCherry construct, psPAX2 packaging construct (Addgene, Plasmid #12260) and pMD2.G envelope construct (Addgene, Plasmid #12259) using Lipofectamine2000 at a 1 to 2.5 ratio in Opti-MEM (Gibco, 31985062). Transfection media was replaced with growth media after 5hrs. Growth media containing the lentivirus was collected after 48hrs, filtered through a Steriflip-GP Filter Unit (Millipore, SCGP00525) and stored at -80^o^C until infection of hFOB1.19 cells.

Freshly thawed hFOB1.19 cells were plated in 100mm TC plates at 2 million cells per plate in triplicate and allowed to adhere for 24hrs. Growth media was replaced with 7mL fresh growth media, 3mL filtered lentivirus, and 5µg/mL Polybrene (Sigma-Aldrich, TR-1003-G). Media was replaced after 72 hrs. and cells were checked for expression of mCherry (see **Supplemental** **Figure 1**). Lentiviral transduction efficiency was estimated using the Countess II FL Automated Cell Counter with visible and Texas Red light cubes (Invitrogen). Cells were split post-infection into cell freezer stocks stored in liquid nitrogen, a 6-well plate for genomic DNA extraction, two 12-well plates for ALP assay, two 6-well plates for RNA extraction, and 100mm TC plates for protein isolation.

**Multiplex Sequencing**

Genomic DNA from CRISPR-edited plates was extracted using the DNeasy Blood and Tissue Kit (Qiagen, 69504) according to kit instructions. Extracted DNA was quantitated using a Nanodrop 2000 (Thermo) and concentrations were normalized to 100ng/µL. The proxy SNP target region was amplified by PCR using 100ng genomic DNA, 10µM forward and reverse PCR primer set, nested primer set, and pooled sequencing primer set (see **Supplemental Figure 2** and **Supplemental Table 1**) with EconoTaq PLUS GREEN 2X Master Mix (Sigma-Aldrich, LUC30033-0) in three concurrent reactions. The final PCR reaction contained pooled sequencing primers approximately 50bp upstream of each CRISPR cut site to provide PCR product sequence for all possible splicing sites. To eliminate primer carry-over, each of the PCR reactions was followed by a purification step using the QIAquick PCR Purification Kit (Qiagen, 28104). Libraries were indexed using NEBNext Multiplex Oligos for Illumina (NEB, E7500S) and NEBNext High-Fidelity 2X PCR Master Mix (NEB, M0541S) for 12 cycles and purified with AMPure XP Beads (Beckman Coulter, A63882) at a ratio of 1:1.8. Libraries were checked on a Bioanalyzer 2100 (Agilent) using the High Sensitivity DNA Kit (Agilent, 5067-4626) for quality then pooled at 4nM. Finally, 10pM of the libraries were sequenced on the MiSeq System (Illumina) using the MiSeq Reagent Kit v3 (600-cycle) (Illumina, MS-102-3003).

Sequencing libraries were first mapped to human genome assembly hg19 using BLAT. The spliced reads were extracted if the splicing junction within the read was larger than 2bp and the rest of the read alignment ratio was above 95%. Spliced reads were further assigned to sgRNA primer pair sequences if the splicing site was within 60bp of the CRISPR-cas9 editing sites. The efficiency of each pair of sgRNAs was calculated independently for each library as the ratio of spliced reads located around their editing sites to total mapped reads. Finally, deletions for each pair of sgRNAs were visualized using ggplot2 (v3.1.0) in R.

**Alkaline Phosphatase Assay**

ALP staining was assessed as described in previous studies with hMSCs^(9)^. Briefly, CRISPR-edited hFOB1.19 cells were seeded in two 12-well plates at 70 thousand cells per well and allowed to adhere for 24hrs. The following day, one plate was moved to 39.5^o^C and allowed to differentiate for 5 days, while the other remained at 33.5^o^C. On the day of the ALP assay, fresh fixation and staining mixtures were prepared from the Alkaline Phosphatase (AP) Leukocyte Kit (Sigma-Aldrich, 86R-1KT). Media was removed from the 12-well plates, and cells were washed twice with DPBS (Mediatech, MT21-031-CV). Cells were fixed in the plate for 1min. according to kit instructions then washed with ultrapure water. Staining solution was applied to the cells and development of color monitored for approximately 20-30min. Once staining was complete, cells were washed with ultrapure water three additional times and allowed to air dry. Plates were photographed and images were converted to grayscale for quantification using Image J software as previously described^(9)^.

**Reverse Transcription-quantitative Polymerase Chain Reaction (RT-qPCR)**

RNA was isolated from CRISPR-edited hFOB1.19 cells using TRIzol Reagent (Thermo, 15596018) after 7 days of differentiation (39.5^o^C). Three-hundred micrograms of RNA was subsequently purified using the Direct-zol RNA Miniprep Plus Kit (Zymol, R2070) according to kit instructions to eliminate any DNA contamination. RNA was converted into cDNA using SuperScript IV VILO Master Mix (Thermo, 11756050), again according to kit instructions. Gene specific primers were obtained from IDT and diluted to 500nM (see **Supplemental Table 1**) for use in qPCR reactions. qPCR was performed on the AriaMx Real-Time PCR Machine (Agilent) using Brilliant III Ultra-Fast SYBR Green RT-qPCR Master Mix (Agilent, 600886) supplemented with ROX reference dye (1:500). Results were exported, normalized to GAPDH and fold change calculated using Cq (ΔR) values and the comparative CT method (ΔΔCT Method) ^(15)^.

**Western Blotting**

Plates seeded with CRISPR-edited hFOB1.19 cells were grown at both 33.5^o^C and 39.5^o^C for 7 days and cells were collected in growth media using cell lifters. Cells were washed in DPBS, collected by centrifugation, then lysed in PD lysis buffer (40mM Tris-HCl, pH 8.0, 500mM NaCl, 0.1% NP-40, 6mM EDTA, 6mM EGTA, 10% Glycerol, 10mM NaF, 1mM Orthovanadate, 1:200 Complete Protease Inhibitor Cocktail (Sigma-Aldrich, 11836170001)) on ice. Supernatants containing whole cell lysates were collected following 5min. centrifugation at maximum speed (21,000 rcf). Total Protein concentration was measured using the Pierce bicinchoninic acid (BCA) Protein Assay Kit (Pierce, 23225) on the SpectraMax iD5 Microplate Reader (Molecular Devices). Samples were normalized to 20 micrograms of whole cell lysate and loaded on NuPage 4-12% Bis-Tris, 1mm Gels (Invitrogen, NP0322). Gels were transblotted onto iBlot 2 Mini Polyvinylidene Fluoride (PVDF) Membrane Stacks (Thermo, IB24002) using the iBlot 2 Gel Transfer Device (Thermo), program P-0. Membranes were blocked in 5% nonfat dry milk and incubated overnight at 4^o^C with EPDR1 antibody (Abcam, ab197932) and α-Tubulin antibody (Santa Cruz Biotechnology, Inc., sc-58666). On the following day, membranes were washed three times with 1X TBST, incubated with Horseradish Peroxidase (HPR) linked secondary antibodies (Santa Cruz Biotechnology, Inc.), washed again, developed with SuperSignal West Pico PLUS Chemiluminescent Substrate (Thermo, 34580) and visualized using the iBright FL1500 Imaging System (Thermo). Quantification of Western immunoblotting bands was performed using the build-in software on the iBright system.
